# Falciform Ligament Inflammation Complicated by Umbilical Vein Thrombophlebitis, Left Portal Vein Thrombosis, and Multiple Hepatic Abscesses: A Case Report

**DOI:** 10.7759/cureus.116585

**Published:** 2026-09-20

**Authors:** Mohammed Bilal Hafiz, Abhilash Mesh, Iona Dixon, Joseph Corlett, Ashleigh Warner

**Affiliations:** 1 General Surgery, Withybush General Hospital, Haverfordwest, GBR

**Keywords:** acute cholecystitis, falciform ligament inflammation, hepatic abscess, pancreatitis, portal vein thrombosis, pylephlebitis, umbilical vein thrombophlebitis

## Abstract

Falciform ligament pathology is a rare cause of acute upper abdominal pain and may resemble more common pancreatic and hepatobiliary disorders. A woman in her 50s presented with sudden severe epigastric pain radiating to her back and marked hyperamylasaemia of 1,747 U/L. This combination fulfilled clinical and biochemical diagnostic criteria for acute pancreatitis, although computed tomography (CT) demonstrated no pancreatic inflammation and instead showed severe inflammatory change centred on the falciform ligament, with associated focal fat infarction, left portal vein thrombosis, and gas locules within segment IV of the liver.

The patient subsequently developed fever, tachycardia, lactic acidosis, and profound hypotension requiring intensive care admission, intravenous antimicrobial treatment, fluid resuscitation, and vasopressor support. Serial CT and magnetic resonance imaging (MRI) demonstrated progressive falciform ligament inflammation, thrombophlebitis of the recanalised umbilical vein, occlusive left portal vein thrombosis, and multiple left-lobe hepatic abscesses. Blood cultures obtained during the febrile deterioration subsequently showed no growth.

She improved with prolonged broad-spectrum antimicrobial therapy and therapeutic anticoagulation. Percutaneous drainage was deferred because she remained clinically stable, her inflammatory markers improved, and the hepatic abscesses remained small and stable on serial imaging. Follow-up CT demonstrated resolution of the hepatic abscesses, reduction in the falciform ligament collection, and recanalisation of the umbilical vein, although the left portal vein thrombosis persisted.

Transabdominal ultrasonography during the index admission demonstrated small gallstones. She was subsequently readmitted with recurrent upper abdominal pain, marked hyperamylasaemia, and CT evidence of acute cholecystitis, with the overall clinical picture considered consistent with gallstone pancreatitis. An outpatient CT four weeks after the second discharge demonstrated resolution of the gallbladder inflammatory changes, while the falciform ligament thickening and fluid density remained stable.

This case demonstrates a rare and potentially life-threatening falciform ligament inflammatory process associated with portovenous thrombosis and hepatic abscess formation in the context of sepsis. The index presentation also met accepted diagnostic criteria for acute pancreatitis, and a concomitant biliary contribution could not be excluded. The case highlights the value of serial cross-sectional imaging, multidisciplinary management, and cautious interpretation of overlapping pancreatic, biliary, and falciform ligament pathology.

## Introduction

The falciform ligament is a double layer of peritoneum extending between the liver, anterior abdominal wall, and diaphragm. Its free inferior border contains the ligamentum teres hepatis, which represents the fibrous remnant of the foetal umbilical vein. Primary inflammatory, ischaemic, thrombotic, and infective disorders affecting these structures are rare.

Falciform ligament appendagitis is a form of focal intra-abdominal fat inflammation or infarction. Patients generally present with epigastric or right upper quadrant pain, and the condition may clinically resemble acute cholecystitis, pancreatitis, peptic disease, or other causes of an acute abdomen. Computed tomography (CT) is central to diagnosis because clinical and biochemical findings are often nonspecific [1,2].

More severe falciform ligament pathology, including necrosis, thrombosis, and abscess formation, has been reported in association with pancreatitis, cholecystitis, cholangitis, portal venous infection, and previous abdominal surgery [2-6]. The anatomical continuity between the ligamentum teres, paraumbilical venous channels, and left portal vein creates a potential route for the spread of infection or thrombosis.

Infective thrombosis of the portal vein or its branches, termed pylephlebitis, may be complicated by bacteraemia, hepatic abscess formation, and systemic sepsis [7]. Unlike uncomplicated falciform ligament appendagitis or isolated focal fat infarction, this case involved falciform ligament inflammation associated with umbilical vein thrombophlebitis, left portal vein thrombosis, multiple hepatic abscesses, and vasopressor-requiring sepsis. This unusual combination of anatomically contiguous inflammatory, thrombotic, and infective findings forms the principal clinical significance of the report. The diagnostic course was additionally complicated by acute pancreatitis at the index presentation, subsequent identification of gallstones, and a later readmission with gallstone pancreatitis.

## Case presentation

A woman in her 50s presented to the Emergency Department with a 12-hour history of sudden, severe epigastric and lower central chest pain radiating to her back. The pain had awakened her from sleep, was constant and crushing in character, and was rated as 8/10 in severity. It was accompanied by nausea and light-headedness but no vomiting, dyspnoea, cough, loss of consciousness, urinary symptoms, or recent travel.

Her medical history included essential hypertension and asthma. Her regular medications included amlodipine, losartan, indapamide, montelukast, inhaled fluticasone/salmeterol, lansoprazole, and intranasal fluticasone. She had been receiving privately prescribed tirzepatide 10 mg once weekly for weight management for approximately 12 months before admission. She was a non-smoker and consumed alcohol occasionally.

On initial assessment, she was visibly uncomfortable but alert and haemodynamically stable. She was afebrile; heart rate was 82 beats/minute, blood pressure was 122/74 mmHg, respiratory rate was 19 breaths/minute, and oxygen saturation was greater than 96% on room air. The abdomen was soft, with epigastric tenderness but no guarding or rebound tenderness. Murphy’s and Rovsing’s signs were negative, and bowel sounds were present. The rest of the systemic examination was unremarkable.

Admission laboratory investigations demonstrated neutrophilic leukocytosis, with a white blood cell count of 17.2 × 10⁹/L and neutrophils of 15.2 × 10⁹/L, despite a C-reactive protein of <5 mg/L (Table 1). There was marked hyperamylasaemia (1,747 U/L), abnormal liver biochemistry, with bilirubin of 25 µmol/L and alanine aminotransferase of 189 U/L, mild hypercalcaemia, with an adjusted calcium of 2.74 mmol/L, and a markedly elevated D-dimer of 3,393 µg/L. Urea and creatinine were within the laboratory reference ranges, and high-sensitivity troponin T was normal.

**Table 1:** Admission laboratory investigations in the emergency department.

Parameter	Result	Units	Reference range
White blood cell count	17.2	×10⁹/L	4.0-11.0
Haemoglobin	144	g/L	115-165
Platelet count	302	×10⁹/L	150-400
Red blood cell count	4.94	×10¹²/L	3.80-5.50
Haematocrit	0.43	L/L	0.37-0.47
Mean cell volume	87	fL	80-100
Mean cell haemoglobin	29.2	pg	27.0-33.0
Red cell distribution width	12.3	%	11.0-14.8
Neutrophil count	15.2	×10⁹/L	1.7-7.5
Lymphocyte count	1	×10⁹/L	1.0-4.5
Monocyte count	0.8	×10⁹/L	0.2-0.8
Eosinophil count	0.1	×10⁹/L	0.0-0.4
Basophil count	0	×10⁹/L	0.0-0.1
Amylase	1,747	U/L	<100
C-reactive protein	<5.0	mg/L	<5
Adjusted calcium	2.74	mmol/L	2.20-2.60
Bilirubin	25	µmol/L	<21
Total protein	74	g/L	60-80
Albumin	44	g/L	35-50
Alkaline phosphatase	115	U/L	30-130
Alanine aminotransferase	189	U/L	<35
Sodium	134	mmol/L	133-146
Potassium	3.7	mmol/L	3.5-5.3
Urea	6.2	mmol/L	2.5-7.8
Creatinine	73	µmol/L	46-92
Estimated glomerular filtration rate	73	mL/min/1.73 m²	-
High-sensitivity troponin T	6	ng/L	<14
D-dimer	3,393	µg/L	<500

Venous blood gas analysis demonstrated a pH of 7.44 and lactate of 2.7 mmol/L. The combination of characteristic severe epigastric pain radiating to the back and serum amylase greater than three times the upper reference limit fulfilled two accepted diagnostic criteria for acute pancreatitis [8-10]. Other differentials included acute coronary syndrome, pulmonary embolism, biliary pathology, and acute aortic disease.

All CT examinations were performed using the Aquilion ONE/PRISM Edition CT system (Canon Medical Systems Corporation, Otawara, Japan). CT pulmonary angiography demonstrated no pulmonary embolism. However, CT of the abdomen and pelvis demonstrated severe inflammatory change involving the falciform ligament and extending into the adjacent anterior fat, with associated focal fat infarction. Multiple small gas locules were present within segment IV of the liver, raising concern for falciform ligament necrosis or infection. There was associated thrombosis of the left portal vein and possible underlying inflammation of the common bile duct. The gallbladder appeared unremarkable, and there was no CT evidence of pancreatic or peripancreatic inflammation, bowel obstruction, perforation, or another acute bowel abnormality.

Analgesia was provided with morphine, intravenous paracetamol, and rectal diclofenac. Empirical intravenous co-amoxiclav, metronidazole, and gentamicin were initially commenced because the imaging findings raised concern for an infected or necrotic falciform ligament process consistent with intra-abdominal infection. Intravenous crystalloid was also administered.

Clinical deterioration and intensive care admission

Later that evening, the patient deteriorated acutely and triggered a medical emergency response with a National Early Warning Score of 8. Her temperature increased to 38.5°C, heart rate to 114 beats/minute, and respiratory rate to 22 breaths/minute. Her blood pressure fell to 77/47 mmHg, although she remained alert and maintained an oxygen saturation of 96% on room air.

Repeat venous lactate was 3.5 mmol/L, and electrocardiography demonstrated sinus tachycardia. A chest radiograph showed no acute abnormality. A urinary catheter was inserted for fluid-balance monitoring, and sepsis management was initiated. Blood cultures were obtained at the time of the temperature spike and subsequently showed no growth.

Following discussions between the emergency medicine, general surgery, haematology, and intensive care teams, she was admitted to the intensive care unit (ICU). The differential diagnoses included acute pancreatitis, left portal vein thrombosis, and falciform ligament necrosis or infection.

Due to acute deterioration with fever, lactic acidosis, and hypotension, antimicrobial therapy was broadened to intravenous piperacillin-tazobactam 4.5 g three times daily and intravenous metronidazole 500 mg three times daily to provide broad enteric Gram-negative and anaerobic coverage in the context of severe intra-abdominal sepsis. Further intravenous crystalloid was administered, and a phenylephrine infusion was commenced to maintain a mean arterial pressure of at least 65 mmHg. Therapeutic anticoagulation with subcutaneous enoxaparin 100 mg twice daily was commenced following haematology advice.

The patient improved rapidly. The phenylephrine infusion was discontinued the following morning after stabilisation of her blood pressure. Repeat venous blood gas analysis showed a lactate of 1.1 mmol/L and a pH of 7.45. Her abdominal pain had improved, and she was transferred from the ICU to the general surgical ward. At transfer, her blood pressure was 116/80 mmHg, heart rate was 101 beats/minute, temperature was 37°C, respiratory rate was 19 breaths/minute, and oxygen saturation was 96% on room air.

Surgical ward course and evolving radiological findings

Following step-down from intensive care, the patient remained under the surgical team until discharge on hospital day 16. She remained clinically stable during this period, and her abdominal pain largely subsided. Daily clinical examination demonstrated only mild epigastric tenderness.

Despite clinical improvement, her C-reactive protein increased to 376 mg/L on hospital day 3. Repeat contrast-enhanced CT on hospital day 4 demonstrated progression of oedema within the falciform ligament and discontinuous enhancement of the recanalised umbilical vein, consistent with worsening venous thrombosis. The distal left portal vein thrombus remained present, while the main and right portal veins remained patent.

The gallbladder wall was diffusely thickened, with oedema in the pericholecystic fat. Thickening and enhancement of the extrahepatic biliary tree raised radiological concern for biliary inflammation. Irregular fluid collections surrounding gas locules were suspicious for evolving abscess formation. The pancreas and peripancreatic fat planes remained within normal limits. Overall, comparison of the initial and hospital day 4 CT examinations demonstrated interval progression of the falciform ligament inflammatory process (Figures 1A-1B).

**Figure 1:**
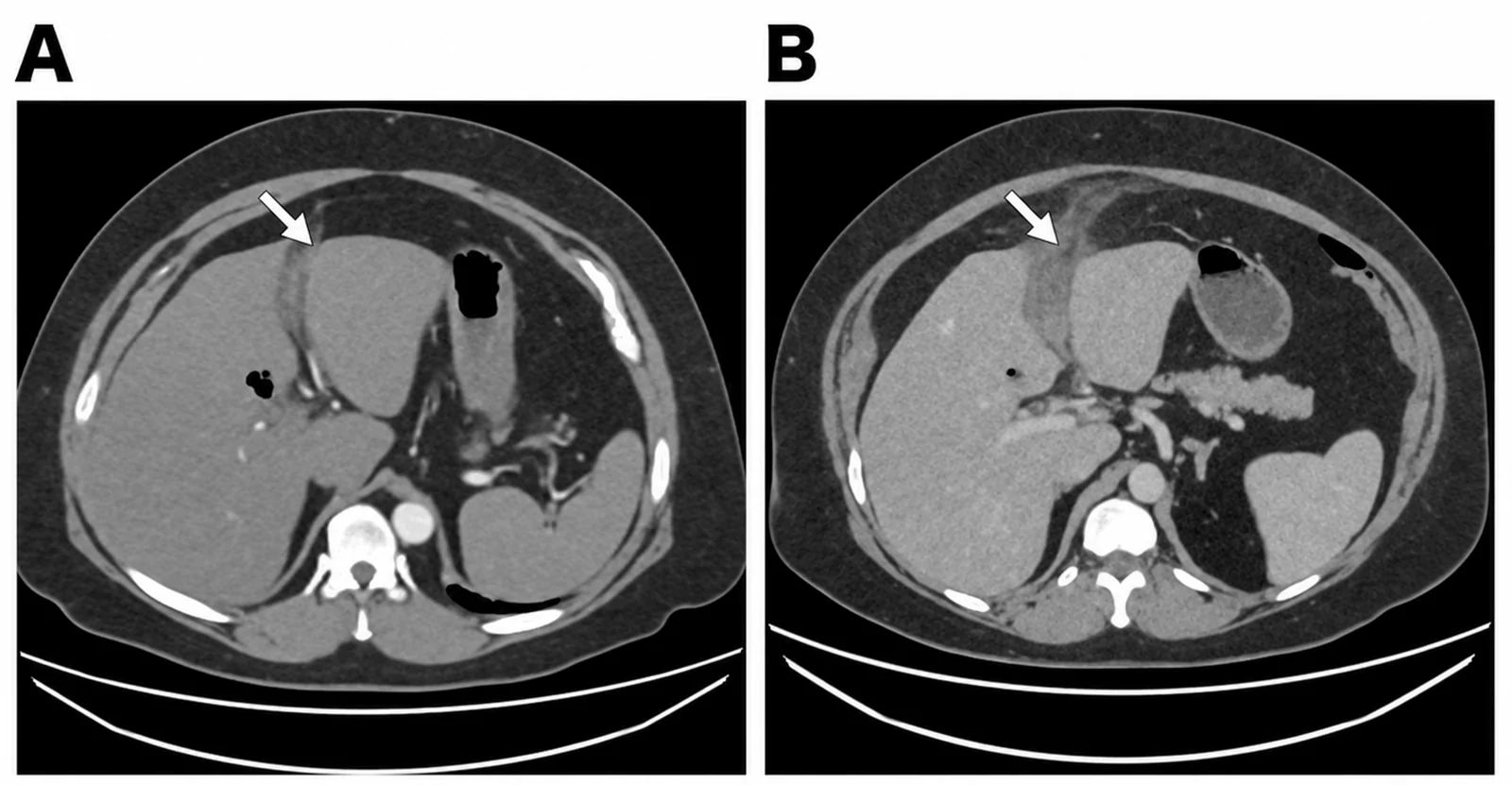
Serial axial contrast-enhanced CT appearances of the falciform ligament inflammatory process. (A) Initial CT on the day of presentation demonstrating inflammatory changes centred on the falciform ligament with adjacent anterior fat stranding. (B) Repeat CT on hospital day 4 demonstrating interval progression of the falciform ligament inflammation, with increased oedema, soft-tissue thickening, and surrounding inflammatory stranding. The white arrows highlight the falciform ligament abnormality in each panel.

The first transabdominal gallbladder and biliary ultrasonography during the index admission was performed on hospital day 7. It demonstrated fatty infiltration of the liver and several hypoechoic, gas-containing areas around the porta hepatis, falciform ligament, and segment IV, the largest measuring approximately 40 mm. Pericholecystic fluid and several subcentimetre gallstones were present, and the common bile duct was of normal calibre. Gallbladder wall assessment was limited because the patient had not fasted.

Contrast-enhanced MRI of the liver was undertaken on the same day using the 1.5-T MAGNETOM Sola system (Siemens Healthineers, Erlangen, Germany). MRI demonstrated multiple abscesses within the left hepatic lobe, the largest measuring 23 mm, an occlusive thrombus involving the left portal vein branch, and associated thrombophlebitis of the umbilical vein within the falciform ligament. Mild diffuse hyperenhancement of the left hepatic lobe was attributed to altered perfusion secondary to left portal vein thrombosis. The hepatic veins and inferior vena cava were patent. There was no intrahepatic or extrahepatic biliary dilatation. The gallbladder appeared normal without wall thickening, and the pancreas demonstrated homogeneous enhancement without focal pathology (Figure 2).

**Figure 2:**
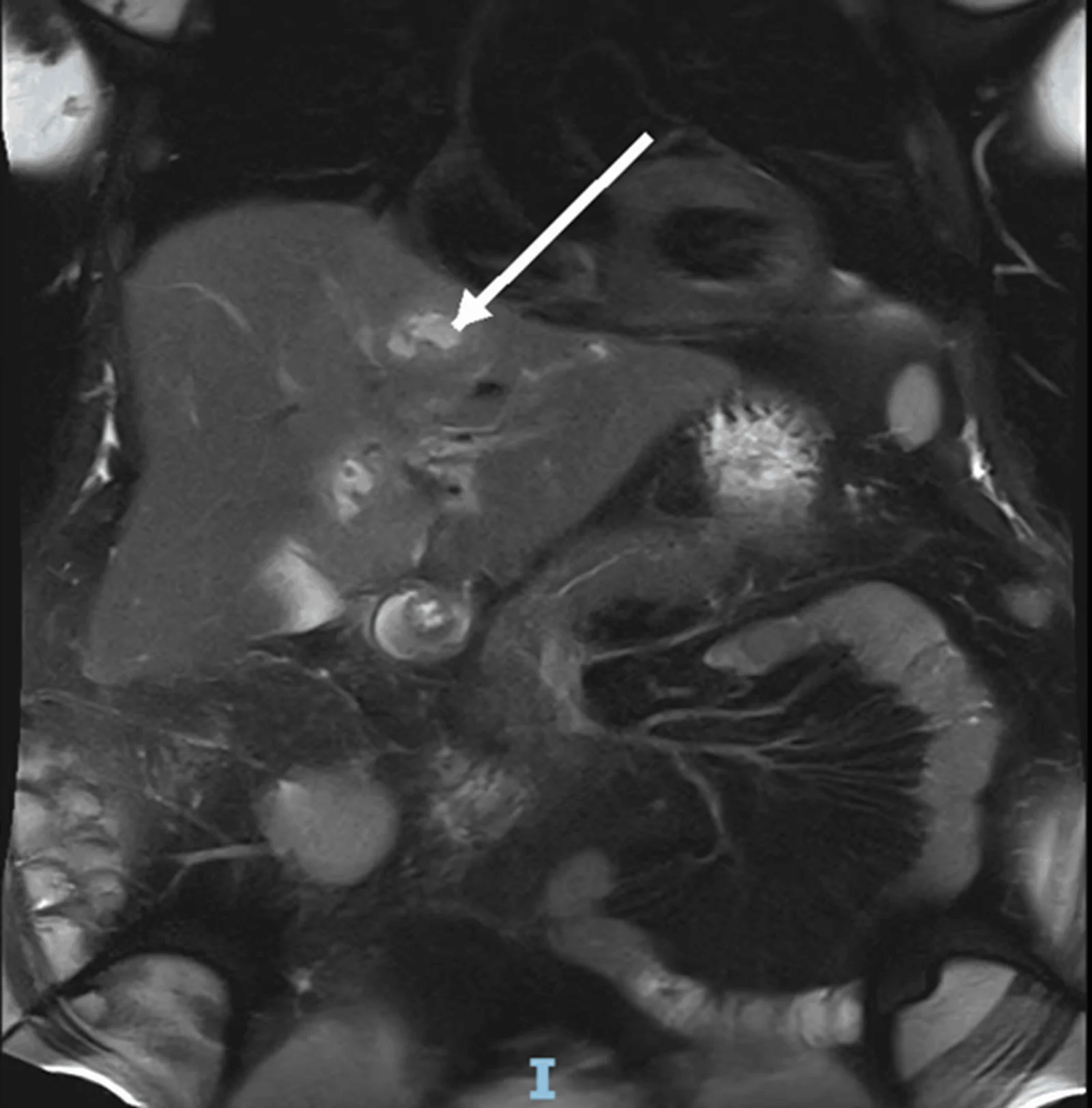
Coronal MRI of the liver on hospital day 7 demonstrating small collections within the left hepatic lobe adjacent to the falciform ligament, consistent with hepatic abscesses. The white arrow displayed on the image highlights one of the collections described that is in keeping with a left hepatic lobe abscess.

The imaging findings supported an inflammatory and infective process involving the falciform ligament, with associated umbilical vein thrombophlebitis, left portal vein thrombosis, and multiple left-lobe hepatic abscesses. The initial CT had demonstrated focal fat infarction and marked falciform ligament inflammation, while the primary infective source remained uncertain.

Following multidisciplinary discussion between the surgical and radiology teams, conservative management with antimicrobial therapy and close clinical and radiological surveillance was continued. Percutaneous drainage was retained as a contingency and was to be reconsidered in the event of recurrent or persistent fever, clinical deterioration, failure of inflammatory markers to improve, or radiological progression of the hepatic abscesses.

Repeat CT on hospital day 11 demonstrated further organisation of the collection in and around the falciform ligament, with formation of a surrounding wall. The collection measured approximately 92 × 32 mm. Importantly, this represented the elongated falciform ligament collection rather than a hepatic abscess; the multiple hepatic abscesses had previously measured up to 23 mm and remained radiologically stable. Although the falciform ligament collection had become more organised, the patient remained clinically well without recurrent fever or haemodynamic instability, and her inflammatory markers were improving on antimicrobial therapy. The imaging was reviewed by a consultant radiologist, who advised continued conservative management without image-guided drainage.

Antimicrobial and anticoagulation treatment

Following ICU admission, intravenous piperacillin-tazobactam and metronidazole were continued for 14 days, until one day before discharge. By completion of intravenous antimicrobial therapy, the C-reactive protein had fallen to 18 mg/L and the white blood cell count to 7.3 × 10⁹/L.

Following discussion with the consultant microbiologist, intravenous treatment was changed to oral amoxicillin 500 mg four times daily, oral metronidazole 400 mg three times daily, and oral co-trimoxazole 960 mg twice daily, all for a further four weeks. The prolonged oral course was selected because of the complicated deep-seated infection, including hepatic abscess formation and associated septic portovenous thrombosis, in the absence of microbiological isolates that would permit narrower organism-directed therapy. Blood cultures remained negative, and no abscess aspirate was available because drainage was not performed; therefore, the infective interpretation was based on the clinical course and serial imaging rather than microbiological confirmation.

Therapeutic enoxaparin 100 mg twice daily was administered for 11 days. Following specialist advice, this was changed to apixaban 10 mg twice daily for seven days, followed by apixaban 5 mg twice daily for a planned treatment duration of six months. Anticoagulation was selected because serial imaging demonstrated occlusive left portal vein thrombosis together with progression of umbilical vein thrombosis. At the time of writing, the patient remained on apixaban 5 mg twice daily as part of this six-month course; no extension beyond six months had been documented.

Persistent mild hypercalcaemia was investigated. Adjusted calcium remained elevated at approximately 2.65 mmol/L despite intravenous saline. Parathyroid hormone was 6.1 pmol/L (reference range 1.6-6.9 pmol/L), inappropriately non-suppressed in the presence of hypercalcaemia; serum protein electrophoresis and urinary Bence Jones protein testing were unremarkable.

The patient was discharged home on hospital day 16 after clinical improvement with prolonged antimicrobial therapy and therapeutic anticoagulation. The hepatic abscesses formed part of an infective process of uncertain primary origin occurring in association with falciform ligament inflammation, umbilical vein thrombophlebitis, and left portal vein thrombosis. The follow-up plan included repeat CT of the abdomen and pelvis following completion of antimicrobial therapy, weekly outpatient blood monitoring, surgical review in clinic, and continuation of apixaban.

Follow-up imaging

Approximately four weeks after discharge, the patient was reviewed in the surgical clinic. She reported that she was well, with no abdominal pain, fever, or vomiting. Her white blood cell count, C-reactive protein, and liver function tests had normalised, although adjusted calcium remained elevated at approximately 2.6 mmol/L.

Follow-up CT performed on the same day demonstrated resolution of both hepatic collections. The falciform ligament collection had reduced from approximately 92 × 32 mm to 57 × 26 mm. The left portal vein thrombosis remained stable. The umbilical vein had recanalised, with no evidence of thrombus propagation. Gallstones were present without gallbladder wall thickening or surrounding inflammatory change (Figure 3).

**Figure 3:**
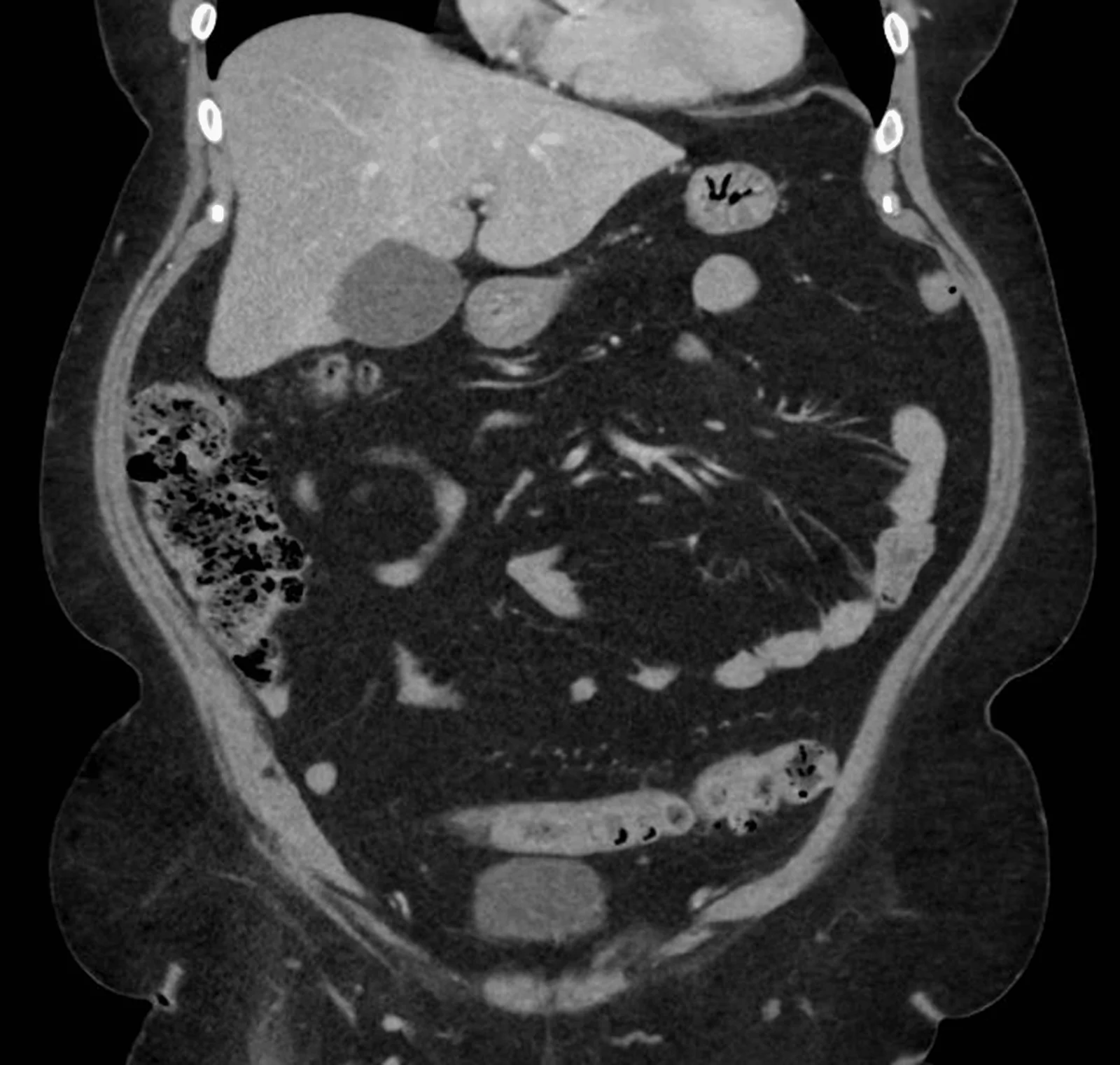
Coronal follow-up contrast-enhanced CT approximately four weeks after the index discharge demonstrating absence of the previously identified left-lobe hepatic abscesses, consistent with interval radiological resolution.

Readmission with gallstone pancreatitis

Eleven days later, the patient was readmitted with a two-day history of upper abdominal pain radiating to her back. Her heart rate was 82 beats/minute, blood pressure was 98/78 mmHg, respiratory rate was 18 breaths/minute, temperature was 36.2°C, and oxygen saturation was 98% on room air. Clinical examination demonstrated moderate epigastric tenderness but was otherwise unremarkable.

Laboratory investigations on readmission demonstrated neutrophilic leukocytosis, with a white blood cell count of 12.4 × 10⁹/L and neutrophils of 9.7 × 10⁹/L, together with a mildly elevated C-reactive protein of 8 mg/L (Table 2). There was marked hyperamylasaemia (1,791 U/L), persistent hypercalcaemia (adjusted calcium 2.78 mmol/L), and elevated lactate dehydrogenase (371 U/L). Liver biochemistry was abnormal, with alkaline phosphatase of 165 U/L and alanine aminotransferase of 230 U/L, while bilirubin remained within the laboratory reference range at 18 µmol/L. Renal function and the measured coagulation parameters were within the displayed laboratory reference ranges.

**Table 2:** Laboratory investigations on readmission in the emergency department.

Parameter	Result	Units	Reference range
White blood cell count	12.4	×10⁹/L	4.0-11.0
Haemoglobin	143	g/L	115-165
Platelet count	335	×10⁹/L	150-400
Red blood cell count	4.87	×10¹²/L	3.80-5.50
Haematocrit	0.41	L/L	0.37-0.47
Mean cell volume	83	fL	80-100
Mean cell haemoglobin	29.3	pg	27.0-33.0
Red cell distribution width	12.6	%	11.0-14.8
Neutrophils	9.7	×10⁹/L	1.7-7.5
Lymphocytes	1.7	×10⁹/L	1.0-4.5
Monocytes	0.5	×10⁹/L	0.2-0.8
Eosinophils	0.3	×10⁹/L	0.0-0.4
Basophils	0.0	×10⁹/L	0.0-0.1
Amylase	1791	U/L	<100
C-reactive protein	8	mg/L	<5
Adjusted calcium	2.78	mmol/L	2.20-2.60
Albumin	44	g/L	35-50
Lactate dehydrogenase	371	U/L	<250
Bilirubin	18	µmol/L	<21
Total protein	72	g/L	60-80
Alkaline phosphatase	165	U/L	30-130
Alanine aminotransferase	230	U/L	<35
Urea	6.7	mmol/L	2.5-7.8
Sodium	136	mmol/L	133-146
Potassium	4.0	mmol/L	3.5-5.3
Creatinine	75	µmol/L	46-92
Estimated glomerular filtration rate	71	mL/min/1.73 m²	-
Prothrombin time	12.3	s	9.0-13.0
Activated partial thromboplastin time	29.0	s	27.0-36.4
Clauss fibrinogen	2.8	g/L	2.0-4.0

Repeat contrast-enhanced CT demonstrated persistent fluid within the falciform ligament fissure with stable adjacent inflammatory change. The gallbladder was acutely inflamed. There were no new peripancreatic inflammatory changes or collections and no evidence of pancreatic necrosis.

Despite the absence of new pancreatic abnormalities on CT, the characteristic upper abdominal pain radiating to the back, marked hyperamylasaemia, abnormal liver enzymes, known gallstones, and radiological evidence of gallbladder inflammation supported a clinical diagnosis of gallstone pancreatitis with concomitant acute cholecystitis.

She was treated with intravenous fluids and antimicrobial therapy, including amoxicillin, metronidazole, and gentamicin. Her C-reactive protein peaked at 204 mg/L before falling to 59 mg/L by discharge. The white blood cell count decreased to 6.2 × 10⁹/L, neutrophils to 3.7 × 10⁹/L, and amylase to 44 U/L. The patient was discharged home after five days with a further nine-day course of oral amoxicillin 1 g three times daily, oral metronidazole 400 mg three times daily, and oral co-trimoxazole 960 mg twice daily.

She was referred to the upper gastrointestinal surgical team for laparoscopic cholecystectomy. At the time of writing, cholecystectomy remained planned, and the patient remained on apixaban 5 mg twice daily as part of the planned six-month course for portal vein thrombosis. The available clinical documentation did not specify whether the timing of cholecystectomy was determined by anticoagulation, residual portal vein thrombosis, or the previous falciform ligament inflammatory process. Persistent hypercalcaemia prompted outpatient neck ultrasonography. This demonstrated a 9 × 4 × 13 mm hypoechoic lesion that could represent a parathyroid adenoma. Further endocrinology assessment was planned.

Subsequent outpatient imaging and summary of clinical course

An outpatient CT abdomen and pelvis was performed four weeks after the second discharge and was compared with the previous study. At the time of this follow-up, the patient remained clinically well, with no acute abdominal pain, fever, or vomiting. The previously noted inflammatory changes involving the gallbladder had resolved. Thickening and fluid density within the falciform ligament fissure, with prominent portal lymph nodes, remained stable, with no interval change. A summary of the clinical timeline is highlighted in Table 3. 

**Table 3:** Timeline of clinical course.

Time point	Key clinical/radiological findings	Management/decision
Index presentation	Severe epigastric pain radiating to the back; amylase 1,747 U/L; WBC 17.2 × 10⁹/L; CT showed falciform ligament inflammation/focal fat infarction, segment IV gas locules, and left portal vein thrombosis.	Analgesia, intravenous fluids, and empirical co-amoxiclav, metronidazole, and gentamicin.
Later same day	Fever 38.5°C, BP 77/47 mmHg, lactate 3.5 mmol/L.	ICU admission, phenylephrine, broadened antimicrobial therapy, and therapeutic enoxaparin.
Hospital day 3	CRP increased to 376 mg/L despite clinical improvement.	Continued antimicrobial therapy and close monitoring.
Hospital day 4	CT showed progressive falciform ligament oedema, worsening umbilical-vein thrombosis, persistent left portal vein thrombosis, and evolving collections.	Continued conservative treatment and anticoagulation.
Hospital day 7	Ultrasound showed subcentimetre gallstones; MRI demonstrated left-lobe hepatic abscesses up to 23 mm, occlusive left portal vein thrombosis, and umbilical-vein thrombophlebitis.	Continued antimicrobial therapy, anticoagulation, and radiological surveillance.
Hospital day 11	Falciform ligament collection organised to approximately 92 × 32 mm; hepatic abscesses remained stable.	Consultant radiology review; image-guided drainage deferred because of clinical and biochemical improvement.
Hospital day 16	CRP 18 mg/L and WBC 7.3 × 10⁹/L; clinically improved.	Discharged on four weeks of oral amoxicillin, metronidazole, and co-trimoxazole; apixaban continued.
~4 weeks after index discharge	Hepatic abscesses resolved; falciform collection reduced to 57 × 26 mm; left portal vein thrombosis stable; umbilical vein recanalised.	Continued outpatient follow-up and anticoagulation.
11 days later	Readmission with recurrent upper abdominal pain, amylase 1,791 U/L, abnormal liver enzymes, and CT evidence of acute cholecystitis.	Managed as gallstone pancreatitis with acute cholecystitis; antimicrobial therapy and supportive treatment.
~4 weeks after second discharge	Gallbladder inflammatory changes resolved; falciform ligament thickening/fluid density stable.	Ongoing surgical and endocrine follow-up; laparoscopic cholecystectomy planned.

## Discussion

Falciform ligament appendagitis is a rare form of focal intra-abdominal fat inflammation or infarction. Reported patients typically present with acute epigastric or right upper quadrant pain, and the condition may be mistaken for gallbladder disease, pancreatitis, or other causes of an acute abdomen. CT may demonstrate heterogeneous increased density of fat adjacent to the falciform ligament, surrounding inflammatory stranding, or a hyperattenuating rim [1,2]. Uncomplicated cases have resolved with analgesia and conservative treatment.

The presented case differed substantially from uncomplicated focal fat infarction. The patient developed sepsis with vasopressor-requiring hypotension, progressive falciform ligament inflammation, thrombophlebitis of a recanalised umbilical vein, occlusive left portal vein thrombosis, and multiple hepatic abscesses. These abnormalities occurred in anatomically contiguous structures and are likely to represent a linked inflammatory, infective, and thrombotic process, although the direction of progression cannot be established.

The precise pathological sequence cannot be established. One possibility is that focal fat infarction and inflammation around the falciform ligament became secondarily infected, followed by extension into the recanalised umbilical vein and left portal vein, with subsequent hepatic seeding and abscess formation. An alternative explanation is that a biliary or hepatic infection produced portal venous infection and left hepatic abscesses, with secondary spread into the umbilical vein and falciform ligament.

The imaging supported elements of more than one possible pathological sequence. The initial scan demonstrated focal fat infarction with marked falciform ligament inflammation and adjacent necrotic or infective change. The following CT raised concern for inflammatory changes involving the gallbladder and extrahepatic biliary tree, with evolving hepatic abscesses. However, MRI later demonstrated no biliary dilatation and a normal-appearing gallbladder.

The later admission with gallstone pancreatitis and CT evidence of acute cholecystitis confirmed clinically significant gallstone disease. This finding also raises the possibility that biliary disease contributed to the index episode. However, follow-up CT after the second admission demonstrated resolution of the gallbladder inflammatory changes, while the falciform ligament thickening and fluid density remained stable. These observations do not establish whether biliary disease initiated the falciform ligament and portovenous process, and the primary infective source therefore remains uncertain.

Falciform ligament abscesses have previously been reported in association with cholecystitis, obstructive cholangitis, pancreatitis, and portal venous infection [3-6]. Warren et al. described a falciform ligament abscess arising from left-sided portal pyaemia following malignant obstructive cholangitis and proposed spread through the paraumbilical veins [5]. Doklestić et al. reported a falciform ligament abscess with left portal vein thrombophlebitis, demonstrating a similar anatomical association to the present case [4].

The combination of intra-abdominal infection, left portal vein thrombosis, umbilical vein thrombophlebitis, hepatic abscesses, and systemic sepsis is compatible with a localised form of pylephlebitis; this is an infective thrombosis of the portal vein or its branches and can complicate infections occurring within areas drained by the portal venous circulation. Blood cultures may be negative, particularly after antimicrobial treatment has commenced, and diagnosis may therefore depend on compatible imaging and clinical findings. Empirical treatment generally requires broad-spectrum coverage of likely enteric organisms and a prolonged course, commonly at least four to six weeks [7].

The patient received prolonged broad-spectrum antimicrobial therapy directed against likely intra-abdominal organisms. Blood cultures obtained during the febrile deterioration showed no growth, and no abscess sample was obtained because drainage was not performed. The absence of microbiological confirmation limits conclusions regarding the primary source and causative organism but does not negate the radiological evidence of hepatic abscesses and portovenous thrombophlebitis.

Drainage or surgical intervention has been used in previously reported falciform ligament abscesses, particularly where a mature collection persisted or the patient failed to respond to antimicrobial therapy [5,6]. In this case, the 92 × 32 mm measurement represented the elongated falciform ligament collection rather than a hepatic abscess; the hepatic abscesses were small (up to 23 mm) and remained stable. Drainage was deferred following consultant radiology review because the patient was clinically improving, had no persistent fever or haemodynamic instability, and demonstrated falling inflammatory markers. Follow-up imaging subsequently confirmed complete resolution of the hepatic abscesses and reduction of the falciform ligament collection, providing retrospective support for the conservative strategy.

The role of anticoagulation in infective portal vein thrombosis is not established by randomised evidence and is generally individualised. It may be considered where thrombosis progresses on imaging, extends within the portal venous system, or persists despite antimicrobial therapy [7]. Anticoagulation was selected in this case because of occlusive left portal vein thrombosis and progression of umbilical vein thrombosis. The subsequent recanalisation of the umbilical vein without thrombus propagation was reassuring, although the left portal vein thrombus remained visible on follow-up imaging.

The index presentation fulfilled accepted diagnostic criteria for acute pancreatitis because the patient had characteristic severe epigastric pain radiating to the back and serum amylase greater than three times the upper reference limit [8-10]. Characteristic pancreatic imaging findings are not required when two diagnostic criteria are present. The repeatedly normal pancreatic imaging therefore does not exclude acute pancreatitis. However, the dominant radiological abnormalities involved the falciform ligament, umbilical vein, left portal vein, and left hepatic lobe, so the relative contribution of pancreatitis to the overall clinical syndrome remains uncertain. Serum lipase was not measured.

A biliary aetiology for the index pancreatitis cannot be excluded. The first transabdominal gallbladder and biliary ultrasound was performed on hospital day 7 and demonstrated several subcentimetre gallstones, pericholecystic fluid, and a normal-calibre common bile duct, although gallbladder wall assessment was limited because the patient had not fasted. Liver biochemistry was also abnormal at presentation, and the patient later experienced gallstone pancreatitis. Current guidelines recommend abdominal ultrasonography of the gallbladder and biliary system during hospitalisation to determine the aetiology of acute pancreatitis [9,10]. Accordingly, concomitant biliary pancreatitis may have coexisted with the falciform ligament-centred inflammatory and infective process, and the initial abdominal pain should not be attributed exclusively to the falciform ligament pathology.

The subsequent admission was attributed to recurrent gallstone pancreatitis because of recurrent characteristic pain, marked hyperamylasaemia, abnormal liver enzymes, known gallstones, and radiological evidence of acute gallbladder inflammation. Interval outpatient CT subsequently confirmed resolution of the gallbladder inflammatory changes.

Tirzepatide was considered as a possible contributor during the initial admission and was discontinued. However, no causal association between tirzepatide and falciform ligament inflammation, portal vein thrombosis, or hepatic abscess formation can be established from this case. The presence of gallstones, later gallstone pancreatitis, and persistent hypercalcaemia provide alternative potential contributors to pancreatic symptoms and hyperamylasaemia. Tirzepatide is therefore best regarded as a relevant medication exposure rather than the proven cause of the presentation.

Persistent hypercalcaemia remained a potential alternative contributor to pancreatitis. A non-suppressed parathyroid hormone level and a possible parathyroid lesion on ultrasonography raised the possibility of primary hyperparathyroidism, although this remained unconfirmed pending specialist endocrine assessment.

The principal limitations of this case are the absence of microbiological confirmation from blood or abscess fluid, absence of histopathological confirmation of the underlying falciform ligament pathology, lack of serum lipase measurement, and uncertainty regarding both the primary source and direction of infective spread. Although gallstones were identified on hospital day 7, no transabdominal gallbladder ultrasound was performed at initial presentation, so an occult biliary contribution to the index pancreatitis cannot be excluded. No thrombophilia screen was known to have been performed. Despite these limitations, serial imaging demonstrated a clear temporal association between falciform ligament inflammation, umbilical vein thrombophlebitis, left portal vein thrombosis, and hepatic abscess formation.

## Conclusions

Falciform ligament pathology should be considered in patients presenting with acute epigastric or right upper quadrant pain when cross-sectional imaging demonstrates focal anterior perihepatic inflammation. Falciform ligament inflammation may be associated with serious contiguous complications, including umbilical vein thrombophlebitis, portal vein thrombosis, hepatic abscess formation, systemic sepsis, and haemodynamic instability; however, the direction of the pathological sequence cannot be established from this case.

This case demonstrates the value of serial CT and MRI when multiple intra-abdominal processes may coexist. The index presentation also fulfilled accepted diagnostic criteria for acute pancreatitis, and because gallstones were identified during the same admission and the patient later developed gallstone pancreatitis, a concomitant biliary aetiology cannot be excluded. Multidisciplinary treatment with prolonged antimicrobial therapy, therapeutic anticoagulation, and close radiological surveillance achieved resolution of the hepatic abscesses without invasive drainage. The case highlights the importance of distinguishing observed associations from causation when falciform ligament, portovenous, pancreatic, and biliary abnormalities overlap.
